# Contrasting effects of historical contingency on phenotypic and genomic trajectories during a two-step evolution experiment with bacteria

**DOI:** 10.1186/s12862-016-0662-8

**Published:** 2016-04-23

**Authors:** Jessica Plucain, Antonia Suau, Stéphane Cruveiller, Claudine Médigue, Dominique Schneider, Mickaël Le Gac

**Affiliations:** Univ. Grenoble Alpes, Laboratoire Technologies de l’Ingénierie Médicale et de la Complexité – Informatique, Mathématiques et Applications (TIMC-IMAG), F-38000 Grenoble, France; Centre National de Recherche Scientifique (CNRS), TIMC-IMAG, F-38000 Grenoble, France; Conservatoire national des arts et métiers, Paris, France; Direction des Sciences du Vivant, CEA, Institut de Génomique, Genoscope & CNRS-UMR8030, Évry, France; Laboratoire d’Analyses Bioinformatiques en Génomique et Métabolisme, Évry, France; Ifremer, DYNECO/Pelagos, 29280 Plouzané, France

**Keywords:** Experimental evolution, *Escherichia coli*, Adaptation, Historical contingency, Epistasis

## Abstract

**Background:**

The impact of historical contingency, i.e. the past evolutionary history of a population, on further adaptation is mostly unknown at both the phenotypic and genomic levels. We addressed this question using a two-step evolution experiment. First, replicate populations of *Escherichia coli* were propagated in four different environmental conditions for 1000 generations. Then, all replicate populations were transferred and propagated for further 1000 generations to a single new environment.

**Results:**

Using this two-step experimental evolution strategy, we investigated, at both the phenotypic and genomic levels, whether and how adaptation in the initial historical environments impacted evolutionary trajectories in a new environment. We showed that both the growth rate and fitness of the evolved populations obtained after the second step of evolution were contingent upon past evolutionary history. In contrast however, the genes that were modified during the second step of evolution were independent from the previous history of the populations.

**Conclusions:**

Our work suggests that historical contingency affects phenotypic adaptation to a new environment. This was however not reflected at the genomic level implying complex relationships between environmental factors and the genotype-to-phenotype map.

**Electronic supplementary material:**

The online version of this article (doi:10.1186/s12862-016-0662-8) contains supplementary material, which is available to authorized users.

## Background

The potential influence of past history on evolutionary outcomes has been debated since Darwin first addressed the issue in the "Origin of Species [1, 2]. It has been popularized after the famous metaphor of Stephen J. Gould about “replaying life’s tape”: “You press the rewind button and, making sure you thoroughly erase everything that actually happened, go back to any time and place in the past… Then let the tape run again and see if the repetition looks at all like the original… Any replay of the tape would lead evolution down a pathway radically different from the road actually taken” [3].

Two main approaches are used to analyze historical contingency. One investigates the level of evolutionary parallelism at the macro-evolution level, i.e. the major innovations that have repeatedly evolved in life history [4] or whether evolution is repeatable in natural populations/species that evolved independently in similar but geographically disconnected environmental conditions, such as oceanic islands or glacial lakes [5]. A famous example involves the repeated adaptive radiations of *Anolis* lizards in Caribbean islands [6]. In a second approach, historical contingency can be investigated by experimental evolution with viruses [7–9], bacteria [10–15], and yeasts [16] that are propagated from an initial ancestral strain in controlled laboratory conditions for hundreds to tens of thousands of generations [17]. Since microorganisms can be cryo-preserved and revived, these evolution experiments provide frozen fossil records that allow comparisons between evolved clones or populations and their common ancestor and therefore investigation of the detailed evolutionary trajectories at both phenotypic and genomic levels.

Several evolution studies have shown that historical contingency may play a major role in the evolution of protein structure and properties [18], as well as of rare phenotypic innovations that required successive mutations and a potentiating genetic background [11, 12]. Hence, the emergence, after a very long evolutionary time (>30,000 generations), of a citrate-utilizing phenotype in *Escherichia coli* was contingent upon mutations accumulated during a particular evolution history [11–14]. Successive and contingent mutational steps were also detected during the co-evolution of λ phage and its *E. coli* host that first allowed evolved bacteria to escape phage infection, and then evolved phages to target a new bacterial membrane receptor, from which newly evolved bacteria escaped again [9]. In another study, the establishment of a stable bacterial polymorphism was found to be contingent upon the complex epistatic interactions between mutations [15]. By contrast, other studies have found that adaptation is not always historically contingent. A two-step evolution experiment with *E. coli* showed similar fitness evolution despite distinct initial genetic state and evolutionary history of the evolved populations [10]. Phenotypic adaptation of Tobacco virus strains to their *N. tabacum* host was also found to be independent from past evolutionary histories [8]. Similarly, yeast populations have been shown to converge at the phenotypic level and partially at the genomic level during adaptation [16].

**Fig. 1:**
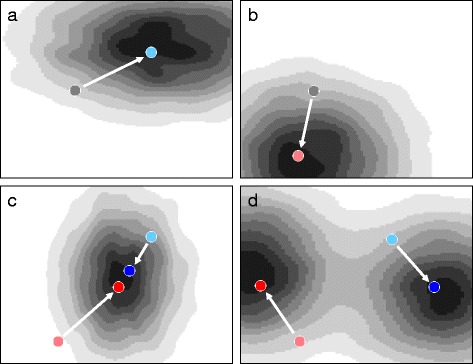
Historical contingency and adaptation in theoretical fitness landscapes. **a** and **b** Adaptive trajectories of a single ancestor (*grey dots*) in two environments represented by two-dimensional fitness landscapes. Light blue (**a**) and light red (**b**) dots indicate the position of a theoretical population after adaptation. **c** and **d** when the two populations resulting from the initial adaptation events are placed in the same environment (light blue and light red dots), historical contingency may cause adaptive convergence (**c**) or divergence (**d**) depending on both the past environment and the fitness landscape of the new environment. Dark blue and dark red dots indicate the position of the populations after adaptation to the new environment. White arrows indicate adaptive trajectories. In the landscapes, fitness increases from white to black

In contrast to the previous studies, we investigated contingency by directly addressing whether and how different past environments influence evolutionary trajectories of a bacterial population at both the phenotypic and genomic levels. In the context of the adaptive fitness landscape metaphor [19–21], populations initiated with the same ancestor and evolving in different environmental conditions will diverge both phenotypically and genetically as they climb the nearest adaptive peaks in their respective environment (Fig. 1a,b). We investigated whether and how such historical adaptive divergence may influence subsequent adaptation to new environmental conditions (Fig. 1c,d). Two scenarios may be considered: in the first, populations with different environmental histories may converge when placed in the same environmental condition, i.e. climb the same fitness peak (Fig. 1c), while in the second, the initial divergence in the historical environments may fuel further divergence in the new environment (Fig. 1d). The topology of the adaptive landscape in the new environment will determine which scenario occurs. If the landscape is relatively smooth with only few adaptive peaks, i.e. few phenotypic/genetic solutions that are mainly additive, then convergence without strong influence of the historical environments would be expected. By contrast, if the landscape is rugged with multiple adaptive peaks, i.e. with multiple phenotypic/genetic solutions that have epistatic interactions, then adaptation to the historical environments would be expected to influence evolution in the new environment, and divergence would be expected between populations that initially evolved in different conditions.

We investigated the influence of evolutionary history on adaptation to a new environment by designing a two-step evolution experiment. Bacterial populations initiated from a single ancestral clone of *E. coli* were first propagated in four different environments (phase I) and then in a single new environment (phase II). Phase I was already performed and analyzed in a previous study, during which we propagated four replicate populations of *E. coli* for 1000 generations in each of four different environments that were different for their carbon sources, structure and oxygenation [22]. These four environments represent the historical environments. We sequenced the genomes of one evolved clone isolated from each of the 16 populations and assayed the growth rate and fitness traits of the 16 clones in all four environments [22]. Here, we performed phase II during which we propagated both a sample from the same 16 populations and a randomly isolated evolved clone from each population in a single new environment for another 1000-generation experiment. Evolved lineages were characterized at both the phenotypic and genomic levels to assess the impact of historical contingency on evolutionary trajectories.

## Results

**Fig. 2:**
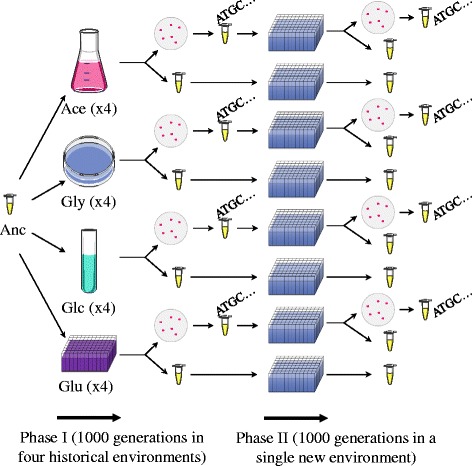
Two-step experimental evolution design. Populations were initiated from a single ancestor (Anc) and propagated in four initial historical environments named Ace (Acetate), Gly (Glycerol), Glc (Gluconate), and Glu (Glucose) during 1000 generations (phase I) [22]. From each replicate population, both a population sample and a randomly isolated evolved clone were preserved and used both for phenotypic assays and to found new populations that were propagated in a single new environment during 1000 generations (phase II). At the end of phase II, a population sample from each of the populations as well as a randomly isolated evolved clone from each of the populations founded from the individual evolved clones derived from phase I were preserved. Genome sequences from the evolved clones sampled after phase I have been previously determined [22] and here we sequenced the genomes the evolved clones sampled after phase II (see Methods)

The impact of historical contingency on evolution was analyzed using a two-step evolution experimental design (described in the Methods section and Fig. 2). Briefly, populations initiated from a single ancestor were propagated in four different environments during 1000 generations [22]. At the end of this phase I, population samples as well as randomly isolated clones (one for each population) were propagated during 1000 additional generations in a final environment (phase II). Growth rate and fitness assays in the final environment were performed on population samples at the end of phases I and II, on clones isolated at the end of phase I, as well as on the population samples derived from these clones at the end of phase II. The populations founded from isolated clones were mainly studied for the genomic aspect of our study. The phenotypic results obtained from the two types of populations were similar (see below).

### Historical contingency and phenotypic evolution

**Fig. 3:**
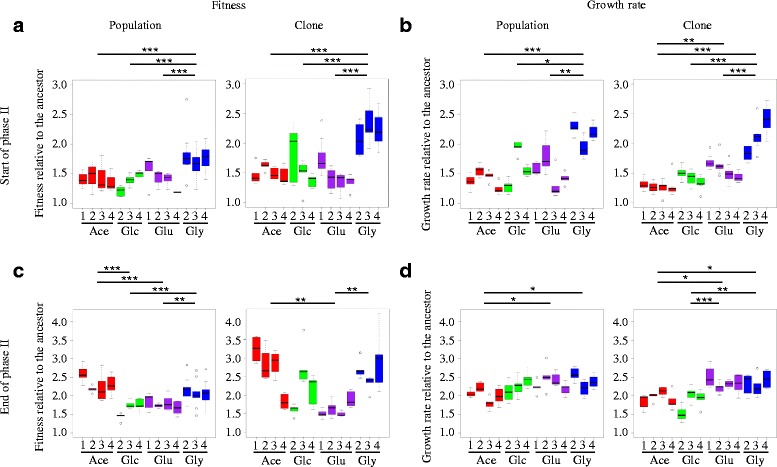
Boxplots indicating the phenotypes (fitness (**a**, **c**) and maximum growth rate (**b**, **d**), both relative to the ancestor) at the start (**a**, **b**) and end (**c**,**d**) of phase II in the final environment for populations founded from population samples (Population) and individual clones (Clone). Results from Tukey HSD post-hoc analyses (ANOVA are presented in Tables S1 and S2) on phenotypic difference between populations that evolved in distinct initial environments are indicated (*< 0.05, **< 0.01, ***<0.001, after Bonferroni corrections)

Divergence of the populations in the four historical environments has been previously reported [22]. Here, we measured the maximum growth rate and fitness of the populations (see Methods) in the new environment at both the start and end of phase II (Fig. 3) to investigate how adaptation in the historical environments affected both the phenotype and evolution trajectories in the new environment. Maximum growth rate and fitness are not independent phenotypic traits. Indeed, fitness often increases owing to growth rate improvement, although this is not systematic. For example, fitness, but not growth rate, may improve if the lag phase is shortened. Conversely, growth rate may improve without dramatic fitness increase if the duration of growth at maximum growth rate is reduced. Analyzing both fitness and maximum growth rate give complementary data on how populations perform and adapt to the final environment.

After phase I (i.e., at the start of phase II), the phenotypic traits of the populations showed differences in the new environment for both population samples and isolated clones (Fig. 3 a,b; Additional file 1: Table S1). Divergence occurred between the populations that evolved in different historical environments (between historical environment divergence, Additional file 1: Table S1, Historical environment effect), but also between the replicate populations that evolved in identical historical environments (within historical environment divergence, Additional file 1: Table S1, Random population effect). The major effect is due to the between historical environment divergence (Additional file 1: Table S1, η^2^) and illustrates that adaptation in different initial environments affected the performance in the new environment. This is especially true for the populations and clones that initially evolved in Gly. These samples tend to display both higher fitness and growth rate in the final environment compared to the population and clones that initially evolved in the three other environments (Fig. 3a,b). To a lesser extent, within environment divergence shows that differences in evolutionary trajectories of replicate populations that evolved in identical historical environments also affected the phenotypic performance in the new environment.

After phase II, the maximum growth rate and the fitness of the populations increased indicating adaptation to the new environment (Fig. 3c,d). This adaptation was strongly dependent upon the historical environment (between historical environment divergence, Additional file 1: Table S2, Historical environment effect). This may be illustrated by the major fitness improvement of the populations that initially evolved in Ace compared to the populations that initially evolved in Glc and Glu (Fig. 3c). One may wonder whether such environment-specific historical contingency may be caused by initial differences in growth rate and fitness. Indeed, both growth rate and fitness improvement rates have been shown to depend on initial levels [23–25]. However, in our experiment, this cannot be the only explanation for the detected contingency, because populations displaying similar fitness levels at the end of phase I (for example those that initially evolved in Ace, Gly, and Glc) have very different fitness levels at the end of phase II (higher fitness for those that initially evolved in Ace). The adaptation during phase II is also influenced by the differences between replicate populations (within historical environment divergence, Additional file 1: Table S2, Random population effect). These results show that historical evolution affected the ability to adapt to a new environment.

**Fig. 4:**
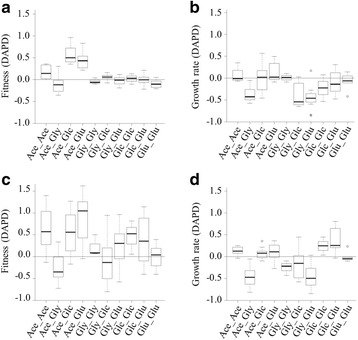
Boxplot showing historical environment-specific divergence or convergence after Phase II. We measured the Difference between the end and start of phase II in the Absolute Phenotypic Difference (DADP, see Methods) for fitness (**a**, **c**) and growth rate (**b**, **d**) between any pair of populations (see Methods). Results are given for populations founded from Phase I population samples (**a**, **b**) and isolated clones (**c**, **d**). Population pairs are grouped based on the historical environments (for example, all comparisons between populations that evolved in Ace and Gly during the first phase are grouped in the Ace_Gly category). DAPD values below and above 0 indicate phenotypic convergence and divergence, respectively, during Phase II

We then wanted to better visualize whether the populations that initially evolved in different environments converged or diverged when adapting to the same final environment. For all pairwise combination of populations and for both fitness and maximum growth rate, we calculated the Difference between the end and start of phase II of the Absolute Phenotypic Difference between the populations (DAPD, see Methods, Fig. 4). Depending on the historical environments, patterns of convergence and divergence (Fig. 4c,d) were identified during phase II. The fitness of the populations that evolved in Ace during phase I diverged from the populations that evolved in Glc and Glu (Fig. 4a,c), due to a major fitness improvement of the populations that initially evolved in Ace during phase II (Fig. 3 a,c). Also, the maximum growth rate of the populations that initially evolved in Ace and Glu, increased during phase II (Fig. 3b,d), leading to the convergence with the populations that initially evolved in Gly (Fig. 4b,d).

### Historical contingency and genetic evolution

**Fig. 5:**
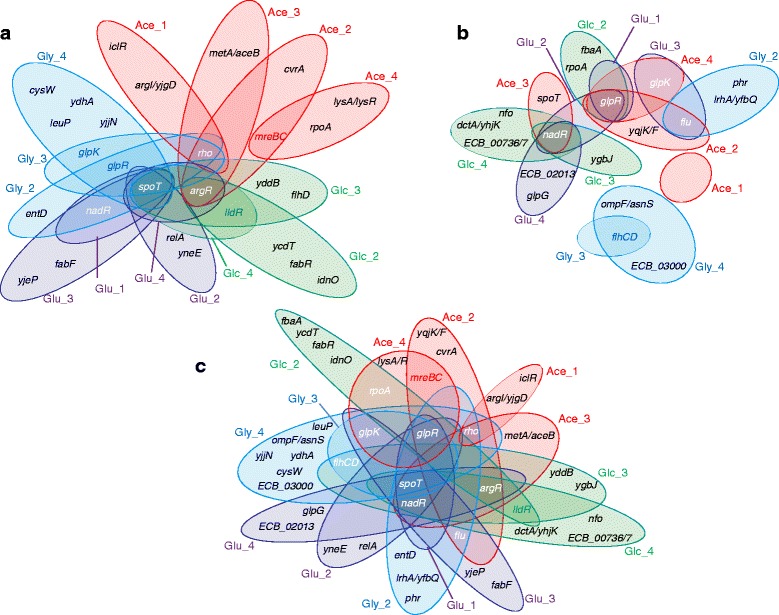
Mutational events in phase I (**a**), phase II (**b**) and both (**c**). The four historical environments are indicated with colours, with red, blue, green and purple corresponding to Ace, Gly, Glc, and Glu, respectively. Genes written in white have been affected by mutations in more than one historical environment. Genes written in colours have been affected by mutations in more than one replicate population from either identical historical environments during phase I or coming from identical historical environments during phase II, the colour corresponding to a given historical environment. Genes specific to the *E. coli* B ancestor strain are indicated with their ECB numbers. In **a**, mutated genes are dependent on the historical environment (Fisher exact test *p* = 0.039). In **b**, mutated genes are not influenced by the historical environment (Fisher exact test *p* = 0.981). In **c**, mutated genes are not influenced by the historical environment (Fisher exact test *p* = 0.562). Statistical tests were performed on genes written in white

In addition, we investigated the impact of historical contingency on the genomic changes that occurred during the two phases. Sequencing the genomes of one evolved clone sampled from each of the populations at the end of phase I (Fig. 5a) allowed us to detect 53 mutations [22]. Here, we sequenced the genomes of one evolved clone sampled from each population at the end of phase II (Fig. 5b, Additional file 1: Tables S3 and S4). We detected 31 new mutations (30 by genome sequencing and one by PCR, see below) including 23 point mutations, 20 of which are in coding regions (two producing stop codons) and three in intergenic regions. We also characterized six small deletions including four in coding regions and two in intergenic regions, one 3-bp insertion and one IS*186* insertion in a coding region. Genetic parallelism was detected as seven genes (*flu*, *rpoA*, *glpR*, *glpG*, *glpK*, *spoT* and *nadR*) carried mutations in clones isolated from independent populations. As genome sequencing may have missed DNA rearrangements, we investigated by PCR amplification and Sanger sequencing whether these seven genes were affected by larger insertions or deletions in the clones where no point mutations were initially identified. We found one additional mutation, an IS*186* insertion in *nadR* in the clone from population Glu_2 (Additional file 1: Tables S3 and S4).

We investigated whether and how the genetic modifications detected after phase II were contingent on the historical environments. After phase I, 53 mutations have been identified with two to seven mutations per clone [22]. After phase II, we found 31 mutations with a lower number of mutations (0 to 4) per clone (two-sided *t*-test, *p* = 0.002, Additional file 1: Tables S3 and S4). During phase I, seven genes (*glpK*, *glpR*, *nadR*, *spoT*, *argR*, *rho* and *lldR*) and one operon (*mreBC*) were repeatedly affected by mutations, i.e. in more than one clone (Fig. 5a). Moreover, the genes mutated in parallel were different from one environment to the other (8 × 4 contingency table Fisher exact test, *p* = 0.0008). This pattern holds even when considering only the four genes (*nadR*, *spoT*, *argR* and *rho*) that were mutated in more than one historical environment (4 x 4 Fisher exact test, *p* = 0.039). By contrast, during phase II 4 genes (*flu*, *glpK*, *glpR* and *nadR*) and 1 operon (*flhCD*) were repeatedly mutated in more than one clone but without historical contingency (Fig. 5b; 5 × 4 Fisher exact test, *p* = 0.728). We observed the same trend when considering all mutations that occurred during both evolution phases (Fig. 5c, 11 × 4 Fisher exact test *p* = 0.108, 9x4 Fisher exact test, *p* = 0.562 when excluding the two environment-specific genes namely *mreBC* and *lldR*). Three additional lines of evidence supported the same conclusion. First, mutations in *glp* genes have been identified after phase I specifically in the clones isolated from the glycerol-containing historical environment (Fig. 5a) and after phase II in various clones irrespective to their historical environments (Fig. 5b), suggesting specific adaptation to glycerol-containing environments. Second, mutations in *nadR* have been identified after phase I in clones from the two glycerol- and glucose-containing environments (Fig. 5a, blue and purple respectively), and after phase II in clones from populations that historically evolved in the two other acetate- and gluconate-containing environments (Fig. 5b, green and pink respectively), suggesting general adaptation. Third, after phase II, one gene (*flu*) was affected by mutations in several clones irrespective to their historical environments, which indicates no strong historical contingency. These clones showed no other shared mutated gene during either phase I or II. We found no evidence for contingency linked to the historical environments on genes mutated during phase II. Likewise, the mutations detected during phase II were not contingent upon the mutations that accumulated during phase I (10 × 5 Fisher exact test *p* = 0.169 considering only those mutations that occurred in parallel during either phase).

## Discussion

We designed a two-step experimental evolution approach to investigate whether adaptation to a given initial (historical) environment may influence further adaption to new environments. We investigated this historical contingency at both phenotypic and genomic levels. Populations that initially evolved during 1000 generations in four different historical environments were transferred to a new environment in which they were propagated for an additional 1000-generation period. Adaptation to the historical environments after phase I was associated with phenotypic as well as genetic divergence when assayed in the new environment. After phase II, the phenotypes of the populations in the new environment were dependent on the historical environments, showing that adaptation was influenced by the environment in which the populations initially evolved. However, the mutations that occurred during phase II were neither contingent on the historical environments nor on the mutations accumulated during phase I.

Our results contrast with others studies showing that historical contingency may be easier to detect at the genomic rather than phenotypic level, especially for fitness-related traits, because selection is supposed to overcome historical contingency [8, 16]. Here, we measured two phenotypes that are obvious targets of selection, maximum growth rate and fitness relative to the ancestor in the new environment. Hence, all populations improved both phenotypic traits in the new environment during phase II, but with a speed/magnitude that was influenced by the historical environments. Moreover, considering growth rate and fitness, convergence or divergence of the populations in the new environment was also dependent on the historical environment. Therefore, even for phenotypic traits that are strongly driven by selection, the evolutionary trajectories of populations adapting to a new environment may be contingent on the environments in which they historically evolved. Eventually, growth rate and fitness may likely improve to the point that they do not appear to be influenced by historical contingency anymore. However, such improvement would have been achieved following different phenotypic evolutionary paths.

It is somehow surprising that the historical contingency detected at the phenotypic level was not related to parallelism at the genomic level. Several hypotheses may explain this discrepancy. First, it is important to emphasize that some mutations, including for example those mediated by the transposition of insertion sequences (IS) and large DNA rearrangements, may have been missed by our genomic analyses. These types of mutations are frequent enough to be drivers of phenotypic evolution, including parallel evolution during experimental evolution [26, 27]. We indeed found one such mutation when we analyzed repeatedly-mutated genes by PCR followed by Sanger sequencing (see Results).

Second, we addressed genomic parallelism at the gene or operon level and assumed that different allelic versions of the same gene resulted in similar phenotypic effects. However, several evolution experiments revealed allelic divergence where different alleles of the same gene may lead to distinct phenotypic traits [15, 28]. In our study, we have no indication that the effects of the different alleles in the repeatedly-mutated genes were more similar between clones that evolved in the same historical environment than in different ones. Indeed, all clones from the same historical environment are only rarely mutated in identical genes and, if so, there is no obvious historical environment-specific pattern. For instance, potential loss-of-function mutations (deletions, frameshifts) in *glpR* were identified in clones from three of the four historical environments and strictly identical *spoT* mutations in clones from two historical environments.

Third, genetic parallelism may occur at a level higher than the gene since mutations of different genes may have similar phenotypic effects, many different genes being involved in identical functions. Although parallelism or convergence at the phenotypic level may be related to mutations in the same genes [17, 29, 30], such phenotypic redundancy may also involve mutations in different genes [31]. In our study, the gene functions give no evidence that mutations occurring in different genes might explain environment-specific historical contingency at the phenotypic level. However, such relationships are impossible to rule out and testing them would require extensive genetic manipulations.

Fourth, the importance of historical contingency may depend on the composition of both the historical and new environments, thereby explaining the seemingly different results obtained in our and previous studies [8, 16]. In the context of environmental contingency, selective pressures may first favour mutations either in environment-specific genes or to compensate maladaptive effects of changes that were beneficial in the historical environments but not in the new one. In the first case, historical contingency may be detected at the phenotypic level during adaptation to a new environment while, in the second case, the initial steps of the adaptive process may mask the phenotypic signature of historical contingency. Here, the selective environment of phase II contains glycerol as the sole carbon source and previous evolution experiments in glycerol-containing medium revealed changes in the genes that are specific to the glycerol metabolism [32]. In our study, ten of the 14 evolved clones carried mutations in glycerol-specific genes at the end of phase II, suggesting mutations directly improving glycerol consumption. Another instance of genetic adaptation in the new environment independent from the historical environments includes the mutations in *flu*, a gene involved in biofilm formation [33], identified in three clones that evolved in three different historical environments. Agitation and thus oxygenation were probably affecting growth in the 1-ml microplates in which all populations were propagated during phase II, thereby potentially favouring clones with different aggregation abilities.

## Conclusions

By designing a two-step evolution experiment with *E. coli*, we investigated whether phenotypic and genomic evolution were contingent on historical environment. At the phenotypic level, evolution in a new environment was contingent on the historical environment even for fitness-related traits (growth rate and fitness relative to the ancestor). However, such historical contingency signatures were not detected at the genomic level. This work illustrates that historical contingency may affect phenotypic adaptation to a new environment without clear genomic signature.

## Methods

### Two-step evolution experiment and strains

The *E. coli* B REL606 strain [34] was used as the ancestor to initiate replicate populations that were propagated during a two-step evolution experiment by daily serial transfers (Fig. 2). During phase I, four replicate populations were propagated in each of four environments (called here historical environments) during 1000 generations, leading to a total of 16 initial populations. Phase I has been performed and analyzed in a previous study [22]. Briefly, all four environments were based on Davis minimal (DM) medium [35]. The first one, named Ace, comprised 15 ml DM supplemented with 2 g/l sodium acetate trihydrate in 50-ml flasks shaken at 200 rpm. The second, named Gly, comprised 15 ml DM supplemented with 1 g/l glycerol in static Petri dishes. The third, named Glc, comprised 15 ml DM supplemented with 1 g/l D-gluconate in test tubes shaken at 200 rpm. The fourth, named Glu, comprised 600 μl DM supplemented with 1 g/l D-glucose in 1-ml 96-well plates shaken at 200 rpm. At the end of phase I, one individual evolved clone was isolated from each of the 16 replicate populations after plating on LB agar plates. Both the population samples and the individual evolved clones were preserved as frozen glycerol suspensions at−80 °C.

During phase II (this work, Fig. 2), 32 populations were propagated in a single evolution environment, hereafter named the new environment, for further 1000 generations. Sixteen populations were initiated with each of the individual evolved clones sampled from each of the 16 replicate populations from the end of phase I, and 16 from each of the population samples. The new environment comprised 600 μl DM supplemented with 1 g/l glycerol in 1-ml 96-well plates shaken at 200 rpm. The 32 populations were propagated at 37 °C with serial daily transfers (24 ^+^/_−_ 2 h) consisting of 300-fold dilutions into fresh medium that allowed ~8.2 [log_2_ (300)] generations per day. Population samples were frozen at−80 °C at 100-generation intervals for all 32 replicate populations. In addition, after 1000 generations at the end of phase II, each of the 16 populations initiated from a single evolved clone was diluted, streaked on LB agar plates and incubated overnight at 37 °C. A single colony was randomly chosen from each plate and frozen at−80 °C. During this step, we observed that two replicate populations initiated from isolated evolved clones, including one that evolved in Gly and the other in Glc during phase I, were contaminated. We therefore discarded these two populations as well as the corresponding replicate populations initiated from population samples from the analyses. Therefore, a total of 28 populations (for each population founded from population samples and isolated clones, four that evolved in Ace during phase I, three that evolved in Gly during phase I, three that evolved in Glc during phase I, and four that evolved in Glu during phase I) were further analyzed.

### Growth assays

Growth profiles were determined with 5-fold replication in the new environment. Each replicate culture was physiologically pre-acclimated by overnight growth in the new environment followed by a 300-fold dilution and 24-h incubation in 96-well plates. Growth profiles were determined by measuring the optical density for each culture at 600 nm (OD_600_) at regular intervals during 24 h. We used the resulting growth curves to compute the maximum growth rate (μ_max_) of each culture (including both evolved population samples and individual evolved clones) relative to their ancestor. Maximum growth rates were measured between 0.2 and 0.8 of the ancestral OD_600_ [22].

### Fitness assays

Fitness assays were performed with 5-fold replication in the new environment by competition experiments as previously described [35]. Briefly, all competitors including the REL606 ancestor and a marked phenotypic variant called REL607 were pre-acclimated in the new environment. The ancestor REL606 and all derived evolved clones are unable to use arabinose as a carbon source (Ara^−^), while REL607 is a REL606 spontaneous revertant that recovered this catabolic ability (Ara^+^). This phenotypic marker has been shown to be neutral [35]. After pre-acclimation, each evolved sample (including both population samples and individual evolved clones) as well as the REL606 ancestral strain as a control were mixed with REL607 at a 1:1 ratio. Mixtures were then diluted 300-fold in fresh medium and incubated for 24 h at 37 °C in the new environment. At days 0 and 1 of each competition experiment, i.e*.* when the two competitors were mixed and after 24 h of incubation respectively, cells were diluted and plated on indicator tetrazolium arabinose (TA) plates. On TA plates, Ara^+^ strains form white pink colonies and Ara^−^ strains red ones [35]. Plates were incubated 24 h at 37 °C and each competitor was scored. Using the initial and final cell counts, we computed the realized (net) population growth of each competitor as G_i_ = ln(C_t1_ x 300/C_t0_), where C_t0_ and C_t1_ are the number of colonies at days 0 and 1 of the competition, respectively, and 300 is the dilution factor. The fitness of one competitor relative to the other was then calculated as the ratio of their net growth rates during the competition experiment: Fitness = G_Ara-_/G_Ara+_, where G_Ara-_ and G_Ara+_ are the realized population growth of the Ara^−^ ancestor or evolved clones and of the Ara^+^ REL607 clone, respectively [35].

### Convergence and divergence

For fitness and growth rate, convergence or divergence between populations during phase II was determined for all pairwise combinations of populations founded with population samples or isolated clones (14 populations each, n(n-1)/2 = 91 combinations each). For each pair of population (α,β) and phenotype (P), the Difference between the end (II) and start (I) of phase II of the Absolute Phenotypic Difference between the populations was calculated as:

$$ {\mathrm{DAPD}}_{\upalpha \upbeta} = \left|{\mathrm{P}}_{\mathrm{I}\mathrm{I}\upalpha}\hbox{-} {\mathrm{P}}_{\mathrm{I}\mathrm{I}\upbeta}\right|\ \hbox{-}\ \left|{\mathrm{P}}_{\mathrm{I}\upalpha}\hbox{-} {\mathrm{P}}_{\mathrm{I}\upbeta}\right| $$

A DAPD < 0 indicates convergence and a DAPD > 0 indicates divergence. The population pairs were grouped based on the historical environments (for example, all comparisons between populations that evolved in Ace and populations that evolved in Gly, during the first phase, are grouped in the Ace_Gly category). As the pairwise comparisons involve pseudoreplication (all the populations are involved in multiple comparisons), no statistical analysis was performed.

### Genome sequencing

The genome sequences of the evolved clones derived from phase I were already available [22]. The genome sequences of the 14 evolved clones isolated after 1000 generations of phase II were determined on the Illumina HiSeq2000 platform (GATC Biotech, Germany) using two lanes of single-ended 76-bp reads. Barcodes were used for each genome to ensure specificity of the reads. Candidate point mutations were identified by comparison with the ancestral genome of REL606 [34] using the PALOMA pipeline [36, 37]. The presence of a mutational event was inferred based on a biological score calculated as the number of reads detecting the mutation over the number of reads covering a given site. For SNPs and Indels, mutations were considered with a biological score > 0.5 and 0.2, respectively. These criteria were chosen after validation of the mutations found by Illumina sequencing because a given mutation was identified either in the evolved clones isolated after both phases I and II (validation of the 53 mutations that occurred during phase I) or after Sanger sequencing for the mutations that were observed during phase II. In addition, 27 mutations with biological scores ranging from 0.06 to 0.47 were not detected using Sanger sequencing. As our work mainly focused on mutations that occurred in more than one clone, i.e. on parallel mutations, seven genes repeatedly affected by mutations (*flu*, *rpoA*, *glpR*, *glpG*, *glpK*, *spoT* and *nadR*) were PCR-amplified for all the isolated evolved clones to detect larger insertions and deletions.

## Ethics

Not applicable.

## Consent to publish

Not applicable.

## Availability of data and materials

The sequences supporting the results of this article are available in the ENA Short Read Archive (PRJEB13018) repository. Mutational events that were identified compared to the reference genome are included within the article and its Additional file 1.

## Additional file

Additional file 1: Table S1. Effect of environmental historical contingency on phenotypes in the new environment at end of phase I (start of phase II).. This table compares the phenotypic traits (maximum growth rate and fitness relative to the ancestor) of the populations in the new environment for both population samples and isolated clones at the beginning of the experiment. Statistical analysis is an Anova test to determine the impact of the historical environment and random population effects on the maximum growth rate and fitness relative to the ancestor of the populations in the new environment at the end of phase I. **Table S2** Effect of environmental historical contingency on adaptation to the new environment at end of phase II. This table compares the phenotypic traits (maximum growth rate and fitness relative to the ancestor) of the populations in the new environment for both population samples and isolated clones at the end of the experiment. Statistical analysis is an Anova test to determine the impact of the historical environment and random population effects on the maximum growth rate and fitness relative to the ancestor of the populations in the new environment at the end of phase I. **Table S3** Mutations by gene identified in the evolved clones sampled from each of 14 populations after both phases I and II. This table gathers all the mutations identified during the two phases of the experiment for each phase and each isolated clone. **Table S4** Mutations identified during phase II. This table details the mutations that occurred during the second phase (nucleotide changes, positions according to the genome sequence of *E. coli* B REL606, amino-acid changes and gene names) for each isolated clone. (PDF 286 kb)

## Abbreviations

Ace: acetate
DAPD: difference of the absolute phenotypic difference between the populations
DM: Davis minimal medium
Glc: D-gluconate
Glu: D-glucose
Gly: glycerol
OD600: optical density at 600 nm
TA: tetrazolium arabinose
