# Supplementary material for: Contrasting effects of historical contingency on phenotypic and genomic trajectories during a two-step evolution experiment with bacteria
Source: BMC Evol Biol. 2016 Apr 23;16:86. doi: 10.1186/s12862-016-0662-8 (PMC4841947; doi:10.1186/s12862-016-0662-8)
Supplement: Additional file 1: Table S1. — Effect of environmental historical contingency on phenotypes in the new environment at end of phase I (start of phase II).. This table compares the phenotypic traits (maximum growth rate and fitness relative to the ancestor) of the populations in the new environment for both population samples and isolated clones at the beginning of the experiment. Statistical analysis is an Anova test to determine the impact of the historical environment and random population effects on the maximum growth rate and fitness relative to the ancestor of the populations in the new environment at the end of phase I. Table S2 Effect of environmental historical contingency on adaptation to the new environment at end of phase II. This table compares the phenotypic traits (maximum growth rate and fitness relative to the ancestor) of the populations in the new environment for both population samples and isolated clones at the end of the experiment. Statistical analysis is an Anova test to determine the impact of the historical environment and random population effects on the maximum growth rate and fitness relative to the ancestor of the populations in the new environment at the end of phase I. Table S3 Mutations by gene identified in the evolved clones sampled from each of 14 populations after both phases I and II. This table gathers all the mutations identified during the two phases of the experiment for each phase and each isolated clone. Table S4 Mutations identified during phase II. This table details the mutations that occurred during the second phase (nucleotide changes, positions according to the genome sequence of E. coli B REL606, amino-acid changes and gene names) for each isolated clone. (PDF 286 kb) [file 12862_2016_662_MOESM1_ESM.pdf]

Table S1 Effect of environmental historical contingency on phenotypes in the new environment at end of phase I (start of phase II)

	SS	η^2	DF	F	*p*-value
GR, Pop, $F_{13,53} = 20.47$; $p = 3.67 \times 10^{-16}$					
Historical Env	0.28	0.51	3	5.29	0.0192
Pop [Historical Env] random	0.18	0.33	10	10.29	2.23×10^{-9}
Residuals	0.09	0.16	53		
Fitness, Pop, $F_{13,63} = 4.81$; $p = 9.89 \times 10^{-6}$					
Historical Env	0.16	0.76	3	9.78	0.0026
Pop [Historical Env] random	0.05	0.10	10	1.59	0.1310
Residuals	0.21	0.13	63		
GR, Clone, $F_{13,133} = 68.28$; $p = 2.20 \times 10^{-16}$					
Historical Env	0.91	0.38	3	24.59	6.23×10^{-5}
Pop [Historical Env] random	0.12	0.12	10	10.60	4.7×10^{-13}
Residuals	0.16	0.50	133		
Fitness, Clone, $F_{13,62} = 8.96$; $p = 5.81 \times 10^{-10}$					
Historical Env	0.36	0.48	3	9.55	0.0028
Pop [Historical Env] random	0.13	0.17	10	3.01	0.0037
Residuals	0.26	0.35	62		

Anova testing the impact of the historical environment, *i.e.* between historical environment divergence (Historical Env), and random population effects, *i.e.* within historical environment divergence (Pop [Historical Env] random), on the maximum growth rate (GR) and fitness relative to the ancestor (Fitness) of the populations in the new environment at the end of phase I. Independent tests were performed for population samples (Pop) and isolated evolved clones (Clone). Maximum growth rates and fitness were log-transformed prior to the analyses; residuals do not deviate from normality.

Table S2 Effect of environmental historical contingency on adaptation to the new environment at end of phase II

	SS	η^2	DF	F	*p*-value
GR, Pop, $F_{13,54} = 5.19$; $p = 6.961 \times 10^{-6}$					
Historical Env	0.06	0.29	3	3.54	0.0558
Pop [Historical Env] random	0.06	0.29	10	3.27	0.0023
Residuals	0.09	0.14	54		
Fitness, Pop, $F_{13,71} = 7.46$; $p = 5.42 \times 10^{-9}$					
Historical Env	0.25	0.44	3	15.76	0.0004
Pop [Historical Env] random	0.05	0.21	10	1.69	0.0993
Residuals	0.22	0.35	71		
GR, Clone, $F_{13,53} = 7.48$; $p = 4.62 \times 10^{-8}$					
Historical Env	0.15	0.48	3	7.02	0.0080
Pop [Historical Env] random	0.07	0.10	10	3.13	0.0033
Residuals	0.12	0.42	53		
Fitness, Clone, $F_{13,53} = 17.39$; $p = 1.1 \times 10^{-14}$					
Historical Env	0.51	0.48	3	4.78	0.0256
Pop [Historical Env] random	0.36	0.34	10	9.29	1.18×10^{-08}
Residuals	0.20	0.19	53		

All experiments, analyses and nomenclature are the same as in Table S1.

Table S3 Changes by gene identified in the evolved clones sampled from each of 14 populations after both phases I and II[1]

	Population													
Gene name[2]	Ace_1	Ace_2	Ace_3	Ace_4	Gly_2	Gly_3	Gly_4	Glc_2	Glc_3	Glc_4	Glu_1	Glu_2	Glu_3	Glu_4
entD					A55T									
phr					**S369***									
ECB_00736/7										**Intergenic**				
ompF/asnS							**Intergenic**							
ycdT								P162A						
fabF													A280V	
cvrA		A61S												
yddB									F306L					
yneE												A168V		
ydhA							V98V							
flhC						**R37L**	**P163T**							
flhD									L21V					
ECB_02013														**Y66D**
nfo										**D89Y**				
lrhA/yfbQ					**Intergenic**									
cysW							F191Y							
ygbJ									**G203G**					
relA												G318C		
lysA/R				Intergenic										
fbaA								**+282Y[3]**						
flu		**L314R**			**L443R**								**G263D**	
yqjK/F		**Intergenic**												
agaE							**G133G**							
argR		R2L	K45T					A55S	K15N	N60D				+12-16EELV[3]
mreC				P230S										
mreB		S10P												
rpoA				E273K				**R317L**						
glpR		**G220D**		**Frameshift**	Δ[4]125 bp	R6C	I49N	**D134Y**			**Frameshift**	**Frameshift**		
glpG														**E91***
dctA/yhjK										**Intergenic**				
lldR								V191A	R244C	W78C				
spoT			**I417T**		P393A	P393L	P393L		T442P	R575L	P393T	P393L	P393L	G207D
rho	G324C	D322Y	M219T		D322Y	G324C								
glpK				**A354V**		A55S	S32L						**G226D**	
fabR								T30N						
metA/aceB			Intergenic											
iclR	A202A													
yjeP													A613A	
argI/yjgD	Intergenic													
idnO								T213P						
yjjN							H62N							
nadR			**G290S**		I211N				**Y294C**	**Frameshift**	Q19*	**IS*186*[5]**	P228L	**K300T**
leuP							+G[3]							

[1] Changes that occurred during phases I and II are shown in grey and blue, respectively. Amino-acid changes are shown for mutations inside genes. Asterisks indicate stop codons. Details of the mutations that occurred during phase I are given in [22], and during phase II in Additional file 1: Table S2.

[2]ECB numbers are given for genes that are specific to the *E. coli* B REL606 ancestral strain [34]. Genes flanking intergenic changes are shown.

[3]+ indicates an insertion.

[4]Δ: deletion.

[5]This mutation was detected by PCR experiments (see Results).

Table S4 Mutations identified during phase II

Population	Mutation[1]	Position[2]	Amino-acid change[1,3]	ECB number	Gene name
Ace_2	T/G	3,000,504	L314R	ECB_02800	*flu*
	A/G	3,183,470		ECB_02969/ECB_02970	*yqjK/F*
	C/T	3,488,548	G220D	ECB_03274	*glpR*
Ace_3	T/C	3,762,006	I417T	ECB_03507	*spoT*
	G/A	4,616,396	G290S	ECB_04266	*nadR*
Ace_4	ΔC	3,488,932	G92A - L129*[4]	ECB_03274	*glpR*
	G/A	4,095,644	A354V	ECB_03811	*glpK*
Gly_2	C/A	721,505	S369*	ECB_00668	*phr*
	ΔTGTTA	2,350,665-2,350,669		ECB_02214/ECB_02215	*lrhA/yfbQ*
	T/G	3,000,891	L443R	ECB_02800	*flu*
Gly_3	C/A	1,956,505	R37L	ECB_01862	*flhC*
Gly_4	C/A	1,004,121		ECB_00933/ECB_00934	*ompF/asnS*
	G/T	1,956,128	P163T	ECB_01862	*flhC*
	C/T	3,214,796	G133G	ECB_03000	*agaE*
Glc_2	+GTA[5]	2,956,153-2,956,155	+282Y[5]	ECB_02756	*fbaA*
	C/A	3,367,985	R317L	ECB_03146	*rpoA*
	C/A	3,488,807	D134Y	ECB_03274	*glpR*
Glc_3	G/A	2,756,898	G203G	ECB_02586	*ygbJ*
	A/G	4,616,409	Y294C	ECB_04266	*nadR*
Glc_4	ΔG	795,316		ECB_00736/ECB_00737	
	G/T	2,202,342	D89Y	ECB_02088	*nfo*
	T/C	3,612,971		ECB_03376/ECB_03377	*dctA/yhjK*
	ΔA	4,616,187	E220D - V226*[4]	ECB_04266	*nadR*
Glu_1	ΔT	3,488,470	H246L - *253D - 275*[6]	ECB_03274	*glpR*
Glu_2	ΔT	3,488,942	I89S - L129*[4]	ECB_03274	*glpR*
	IS*186*[7]	4,616,044	D173I - Y186*[4]	ECB_04266	*nadR*
Glu_3	G/A	3,000,351	G263D	ECB_02800	*flu*
	C/T	4,096,028	G226D	ECB_03811	*glpK*
Glu_4	A/C	2,122,430	Y66D	ECB_02013	
	C/A	3,489,783	E91*	ECB_03275	*glpG*
	A/C	4,616,427	K300T	ECB_04266	*nadR*

[1]Δ: deletion.

[2]Positions are given according to the genome sequence of E. coli B REL606 [34].

[3]*: stop codon.

[4]These frameshift mutations resulted in truncated proteins. The first amino-acid that was changed and the amino-acid position where a stop codon was inserted are indicated.

[5]+ indicates an insertion.

[6]This frameshift mutation resulted in a longer protein. The first amino-acid that was changed, the stop codon that was changed into an amino-acid and the amino-acid position where a stop codon was inserted are indicated.

[7]This mutation was detected by PCR experiments (see Results).
